# Supplementary material for: Biosynthetic diversity and antifungal potential of soil-derived Saccharomonospora strains
Source: Appl Environ Microbiol. 2026 Jul 28;92(8):e00765-26. doi: 10.1128/aem.00765-26 (PMC13488351; doi:10.1128/aem.00765-26)
Supplement: Supplementary file 1 [file aem.00765-26-s0001.docx]

**SUPPLEMENTARY MATERIAL**

**Biosynthetic diversity and antifungal potential of soil-derived *Saccharomonospora* strains**

Hildah Amutuhaire^a, b^, Michael Dubovis^c^, Tal Luzzatto-Knaan^c^, Jonathan Friedman^d^, Eddie Cytryn^a^ #

^a^	Institute of Soil, Water and Environmental Sciences, Agricultural Research Organization, Rishon LeZion, Israel.

^b^ Department of Plant Pathology and Microbiology, The Robert H. Smith Faculty of Agriculture, Food and Environment, The Hebrew University of Jerusalem, Rehovot, Israel.

^c^ Department of Marine Biology, The Charney School of Marine Sciences, University of Haifa, Haifa, Israel.

^d^ The Institute of Environmental Sciences, The Hebrew University of Jerusalem, Rehovot, Israel.

#Address correspondence to Eddie Cytryn, eddie@volcani.agri.gov.il

**Supplementary Table 1.** General genomic features of *Saccharomonospora* strains used in this study.

| **Strain_Name** | **BioProject Accession** | **Assembly Accession** | **Size (Mb)** | **GC%** | **# of Scaffolds** | **Total Gene Count** | **Completeness %** | **Contamination%** | **Isolation source grouping** |
| --- | --- | --- | --- | --- | --- | --- | --- | --- | --- |
| *Saccharomonospora amisosensis* DSM 45685 ^T^ | PRJNA546941 | GCF_011761185.1 | 5.53 | 68.5 | 3 | 5407 | 98.33 | 2.17 | Aquatic |
| *Saccharomonospora marina* XMU15 ^T^ | PRJNA61991 | GCF_000244955.1 | 5.97 | 69 | 1 | 5776 | 99.41 | 0.15 | Aquatic |
| *Saccharomonospora piscinae* KCTC 19743 | PRJNA544002 | GCF_005862235.1 | 4.90 | 71 | 11 | 4553 | 99.72 | 0.79 | Aquatic |
| *Saccharomonospora piscinae* CNQ490 | PRJNA169795 | GCF_000527075.1 | 4.94 | 71 | 25 | 4593 | 99.72 | 0.15 | Aquatic |
| *Saccharomonospora glauca* K62 ^T^ | PRJNA63505 | GCF_000243395.2 | 4.56 | 69 | 2 | 4364 | 99.4 | 0.15 | Compost |
| *Saccharomonospora halophila* 8 ^T^ | PRJNA72359 | GCF_000383775.1 | 3.69 | 71 | 116 | 3868 | 73.92 | 0.3 | Hypersaline soil |
| *Saccharomonospora iraqiensis* subsp. *Iraqiensis* IQ-H1 ^T^ | PRJNA72401 | GCF_000430445.1 | 3.90 | 71.5 | 139 | 4101 | 85.23 | 0.69 | Hypersaline soil |
| *Saccharomonospora iraqiensis* subsp. *paurometabolica* YIM 90007 ^T^ | PRJNA61823 | GCF_000231035.2 | 4.67 | 72 | 82 | 4850 | 97.44 | 0.15 | Hypersaline soil |
| *Saccharomonospora saliphila* YIM 90502 ^T^ | PRJNA66541 | GCF_000383795.1 | 4.03 | 71 | 145 | 4091 | 85.08 | 1.05 | Hypersaline soil |
| *Saccharomonospora xinjiangensis* PNSac2 | PRJNA1144073 | GCF_041204665.1 | 4.78 | 69 | 305 | 4464 | 99.27 | 0.84 | Plant seed |
| *Saccharomonospora azurea* NA-128 ^T^ | PRJNA62037 | GCF_000231055.2 | 4.76 | 70 | 1 | 4534 | 96.93 | 0.57 | Soil |
| *Saccharomonospora cyanea* NA-134 ^T^ | PRJNA61997 | GCF_000244975.1 | 5.41 | 69.5 | 1 | 5193 | 99.42 | 0.77 | Soil |
| *Saccharomonospora viridis* JCM 3315 | PRJNA260873 | GCF_000787535.1 | 4.30 | 67.5 | 13 | 3957 | 99.48 | 0.25 | Soil |
| *Saccharomonospora xinjiangensis* 31sw | PRJNA523228 | GCF_004519465.1 | 4.71 | 69 | 1 | 4402 | 98.75 | 0.3 | Soil |
| *Saccharomonospora xinjiangensis* XJ-54 ^T^ | PRJNA62221 | GCF_000258175.1 | 4.78 | 69 | 2 | 4405 | 99.31 | 0.15 | Soil |
| *Saccharomonospora viridis* R81 | PRJNA1307827 | SAMN50675987 | 4.39 | 67.2 | 1 | 4040 | 100 | 0 | Soil |

**Supplementary Table 2.** MetaboScape 2023b processing parameters.

| Intensity threshold [counts] | 10000 |
| --- | --- |
| Minimum peak length [spectra] | 10 |
| Minimum peak length (recursive) [spectra] | 7 |
| Minimum # Features for Extraction | 4/17 |
| Presence of features in minimum # of analyses | 4/17 |
| Lock mass calibration | false |
| Mass calibration | true |
| Calib. RT start | - |
| Calib. RT end | - |
| Filter by sample group | Samples |
| Minimal value of features in sample group | 75% |
| Area | true |
| Primary Ion | [M+H]^+^ |
| Seed Ions | [M+Na]^+^; [M+K]^+^; [M+H_2_O]^+^ |
| EIC correlation | 0.75 |
| Mass range: Start [m/z] | 50 |
| Mass range: End [m/z] | 2500 |
| Retention time range: Start [min] | 0.3 |
| Retention time range: End [min] | 14 |
| Perform MS/MS import | false |
| Group by collision energy | false |
| MS/MS import method | Average |

**Supplementary Table 4**. Chemical classification and averaged intensities of compounds shared between *S. viridis*-R81 and *S. xinjiangensis*

| **Compound_ID** | ***S.viridis*-R81** | ***S.xinjiangensis*** | **Compound pathway** | **Compound superclass** | **Compound class** |
| --- | --- | --- | --- | --- | --- |
| 2677 | 37.088 | 830.766 | Alkaloids | Pseudoalkaloids | Phenylalanine-derived alkaloids |
| 2632 | 164.975 | 5741.968 | Alkaloids | Pseudoalkaloids | Pyridine alkaloids |
| 2141 | 364.906 | 102.944 | Alkaloids | Small peptides | Dipeptides |
| 2847 | 545.456 | 19.498 | Alkaloids | Lysine alkaloids | Quinolizidine alkaloids |
| 1722 | 11.904 | 935.408 | Alkaloids | Lysine alkaloids | Quinolizidine alkaloids |
| 21427 | 11961.758 | 54.860 | Alkaloids | Lysine alkaloids | Pyridine alkaloids |
| 2415 | 89.977 | 1170.591 | Alkaloids | Peptide alkaloids | Piperidine alkaloids |
| 1762 | 12.372 | 6819.871 | Alkaloids | Pseudoalkaloids | Purine alkaloids |
| 1606 | 1795.992 | 27.248 | Alkaloids | Guanidine alkaloids | Tricyclic guanidine alkaloids |
| 1914 | 43.122 | 8472.966 | Alkaloids | Peptide alkaloids | Dipeptides |
| 1143 | 1730.116 | 129.365 | Amino acids and Peptides | Small peptides | Tripeptides |
| 2627 | 519.469 | 11.383 | Amino acids and Peptides | Small peptides | Tripeptides |
| 5980 | 5.490 | 2744.176 | Amino acids and Peptides | Ornithine alkaloids | Polyamines |
| 1598 | 465.592 | 25.327 | Amino acids and Peptides | Small peptides | Tripeptides |
| 1856 | 5.461 | 1289.084 | Amino acids and Peptides | Small peptides | Dipeptides |
| 2115 | 526.513 | 11.273 | Amino acids and Peptides | Small peptides | Tripeptides |
| 1534 | 736.429 | 348.937 | Fatty acids | Fatty esters | Lactones |
| 1034 | 13.155 | 633.079 | Fatty acids | Fatty acyls | Fatty alcohols |
| 1570 | 8.172 | 440.691 | Fatty acids | Fatty acyls | Fatty alcohols |
| 1873 | 69.713 | 2612.186 | Fatty acids | Small peptides | Dipeptides |
| 1865 | 6.590 | 1248.520 | Polyketides | Small peptides | Open-chain polyketides |
| 2051 | 7.060 | 1825.640 | Polyketides | Linear polyketides | Open-chain polyketides |
| 22978 | 649.928 | 11.959 | Shikimates and Phenylpropanoids | Coumarins | Flavanones |
| 1546 | 164.018 | 1193.264 | Terpenoids | Sesquiterpenoids | Irregular monoterpenoids |
| 1566 | 1195.677 | 28.283 | Terpenoids | Steroids | Pregnane steroids |

**Supplementary Table 5.** Mean BGC novelty scores of *Saccharomonospora* genomes

| **GenomeID** | **mean_novelty_score** | **No. of GCFs** |
| --- | --- | --- |
| *S.amisosensis*.DSM.45685 | 0.958 | 14 |
| *S.azurea*.NA-128 | 0.882 | 11 |
| *S.cyanea*.NA-134 | 0.946 | 14 |
| *S.halophila*.8 | 0.972 | 9 |
| S.iraqiensis.IQ-H1 | 0.958 | 6 |
| *S.iraqiensis*.YIM.90007 | 0.977 | 11 |
| *S.marina*.XMU15 | 0.966 | 17 |
| *S.piscinae*.CNQ490 | 0.899 | 21 |
| *S.piscinae*.KCTC.19743 | 0.947 | 18 |
| *S.saliphila.*YIM.90502 | 0.891 | 11 |
| *S.viridis*.JCM.3315 | 0.829 | 9 |
| *S.viridis*.R81 | 0.829 | 9 |
| *S.xinjiangensis*.31sw | 0.942 | 13 |
| *S.xinjiangensis*.XJ-54 | 0.946 | 14 |

**Supplementary Figure 1**. Metabolomic differentiation and molecular networking of three *Saccharomonospora* strains. (A) PCA of LC–MS/MS features (n=3/strain), *S. cyanea* (green), *S. viridis* R81 (orange), and *S. xinjiangensis* (blue). (B) GNPS molecular network of all metabolites identified in the three strains, colored by CANOPUS-predicted classes.
